# Editorial: Prevention and management of non-communicable diseases (NCDs), particularly in COVID-19 response

**DOI:** 10.3389/fpubh.2023.1104758

**Published:** 2023-03-07

**Authors:** Rona Macniven, Sophia Lin, Blessing Akombi-Inyang, Cheng Cheng, Xiaoyue Xu

**Affiliations:** ^1^School of Population Health, UNSW Faculty of Medicine and Health, UNSW Sydney, Sydney, NSW, Australia; ^2^UNSW Faculty of Medicine and Health, UNSW Sydney, Sydney, NSW, Australia; ^3^School of Nursing, Fudan University, Shanghai, China

**Keywords:** risk factors, cardiovascular disease, chronic diseases, pandemics, social determinants of health (MeSH)

Non-communicable diseases (NCDs) comprise many diverse chronic diseases including cardiovascular disease, cancer, respiratory disease, and diabetes (1, 2). NCDs are leading causes of death globally and their contemporary contributing risk factors include high blood pressure, high body mass index, high fasting plasma glucose, tobacco smoking, air pollution and drug use (3). Further, the burden of NCDs is not being experienced equally, with serious and widening gaps in risk factor levels, disease incidence, treatment, and survival between and within different populations (4). Addressing disparities both between-country and within-country globally is critical to reducing the burden of NCDs.

These disparities are partly due to inequities in the social determinants of health and wellbeing (5). The COVID-19 pandemic has reinforced the need to ensure health and social equity. While health authorities globally have reoriented resources and efforts to contain the pandemic, there is concern that the shifting of health, social and economic priorities will have long-lasting detrimental effects on NCD risk, especially among those experiencing inequality. The disruption caused by the COVID-19 pandemic has and will continue to have impacts on risk factor development, screening, treatment and management. Therefore, novel and creative ideas to address social disparities in the current climate is critical to prevent and reduce the burden of NCDs.

This Research Topic presents multidisciplinary research that focus on the impact of the long-term and cumulative effects of risk factors on NCD prevention and management, and the COVID-19 pandemic response relevant to NCD risk factors, access to screening and treatment.

Many countries have developed national strategies for the management and prevention of NCDs (Gassner et al.). These strategies differ in their degree of organization, structure, and implementation across countries. Despite these differences, the strategies mainly aim to prevent NCDs, improve health and quality of life, strengthen self-management and health literacy, reduce health inequalities, and provide integrated care and coordinated services for chronic conditions. However, some strategies remain rather superficial without mentioning specific measures to be carried out to reach the strategy's aims. Therefore, actionable recommendations for the development and implementation of national strategies may consider a life-cycle approach and particularly include aims addressing prevention of NCDs in children and adolescents. In addition, strategies need to be regularly evaluated using appropriate methods to measure target achievement.

Disease control measures used by authorities have had effects on dietary patterns in population. Food insecurity, increased intake of energy dense low nutritional value foods and non-hungry eating resulting from stress and anxiety, coupled with reduced physical activity and increased sedentarism, have contributed to weight gain in a large proportion of populations including in China (Xu et al.). However, for those who were able to maintain nutritious diets, weight gain can be prevented. The epidemic of obesity and related NCDs occurring before the pandemic has been exacerbated by the effects of COVID-related measures and efforts to reduce the NCD burden is being overshadowed. Continued emphasis on promoting and supporting populations to achieve food security and nourishing diets is essential in reducing the NCD burden.

Medication is extremely important for NCDs management. In late 2020, the European Network to Advance Best practices and technoLogy on medication adherencE (ENABLE) conducted a survey across 38 countries, aimed to evaluate the medication management practices in place for NCDs during the COVID-19 pandemic (Ágh et al.). Disparities in ensuring medication management services for NCDs across Europe were highlighted. For instance, only 41% European countries were aware national guidelines regarding maintaining medication availability for NCDs, or advice for patients on how to ensure access to medication and adherence. While electronic prescriptions were available in 92% of countries, online ordering was only available in 46%; and home delivery of prescription medication was only available in 67% of countries.

The impacts of COVID-19 and its related complications are greater and more complex for chronic respiratory diseases such as cystic fibrosis. In France, a national questionnaire, supplemented with interviews, during the first lockdown period in 2020 asked cystic fibrosis patients about their access to healthcare access, anxiety and depression, NCD risk factors including smoking, alcohol, drug consumption and adherence to treatment (Oubaya et al.). While there was minimum impact on patient treatment and care during this time, patients experienced anxiety and depression. Further, patients of lower socioeconomic status experienced negative behavioral risk factor changes such as lower physical activity.

The COVID-19 pandemic is a global health crisis. A growing number of studies have documented the epidemiology, risk factors, and health impacts of COVID-19. Among them, no consensus was reached regarding the determinants of COVID-19 mortality. A path analysis using Our World in Data (OWD) involving 117 countries found that old age and NCD comorbidity are two predominant determinants of COVID-19 deaths, suggesting policymakers and healthcare professionals should further prioritize vaccines and prevention approaches for those two populations (Goswami et al.). Their findings also highlighted how the pandemic caused catastrophic impacts in South America and Europe, followed by North America when compared to Oceania.

This collection of international research across diverse health conditions and behavioral risk factors collectively demonstrates how the impacts for NCDs need to be considered as part of the COVID-19 pandemic response. The often detrimental effects of COVID-19 pandemic restrictions on NCD risk factors, disease management and the resulting future NCD burden must be understood and be integrated in proportionate responses to both the COVID-19 pandemic and possible future pandemics.

## Author contributions

All authors listed have made a substantial, direct, and intellectual contribution to the work and approved it for publication.
